# Supplementary material for: Are carbon emissions trading and green financial instruments synergistic? -Comprehensive quantitative research based on content analysis
Source: PLoS One. 2024 Mar 7;19(3):e0298601. doi: 10.1371/journal.pone.0298601 (PMC10919601; doi:10.1371/journal.pone.0298601)
Supplement: S1 Data — (ZIP) [file pone.0298601.s001.zip › Data/地级市Z_F工作报告.zip!/╣π╬≈/┴°╓▌╩╨/└┤╫╘╣1⁄2╓┌║┼í╛┬φ┐╦╩2╛▌═°í┐_2018.docx]

各位代表：

　　现在，我代表市人民政府向大会作工作报告，请予审议，并请各位政协委员和列席会议的同志提出意见。

　　2017年工作回顾

　　过去的一年，面对错综复杂的发展环境，我们在以习近平同志为核心的党中央坚强领导下，认真学习宣传贯彻党的十九大精神，深刻学习领会习近平新时代中国特色社会主义思想，落实广西“三大定位”新使命"和“五个扎实”新要求#，按照自治区党委、政府和市委的决策部署，深入实施“实业兴市，开放强柳”战略，全力打好“三大攻坚”，营造“三大生态”，加快经济升级、城市转型，促进了经济持续健康发展和社会和谐稳定。

　　——经济发展质量稳步提升。初步统计，全市实现地区生产总值2793.3亿元，增长7%；规模以上工业增加值1344.2亿元，增长5.5%；固定资产投资2689.4亿元，增长15%；财政收入403.8亿元，增长9.1%；一般公共预算收入179.78亿元，增长13%；一般公共预算支出374.07亿元，增长10.17%；非税收入占一般公共预算收入比重28.7%，财政质量进一步提高；全市社会消费品零售总额1149.6亿元，增长10%。经济发展稳中有进、稳中提质，柳州再次获得国务院稳增长督查表彰。

　　——产业转型升级步伐加快。全市全部工业总产值突破5千亿元，达5077亿元，增长8%；工业利税增长13.6%，工业利润增长38.7%。全市新培育2家国家级、4家自治区级工业设计中心，新认定14家自治区级企业技术中心；新增96家规模以上工业企业，达到815家；新增79家高新技术企业，达到280家。战略性新兴产业保持两位数增长，产值占全市工业产值比重12%。万元工业增加值能耗下降1.6%。全市第三产业增加值突破千亿大关，达1073.5亿元，增长11%，占地区生产总值比重提高0.6个百分点，三次产业结构为6.8：54.8：38.4。“两化融合”发展$指数位居全区第一。制造业质量竞争力指数84.22，高于全国平均水平，柳州成为广西首个全国质量强市示范城市。

　　——重点项目投资建设加速推进。实施64项自治区层面重大项目建设，圆满完成自治区下达投资目标。开工170个市级层面重大项目、竣工86个市级层面重大项目，全年重大项目完成投资844.3亿元，增长14.1%。柳梧、三柳、桂三高速公路建成通车，柳州港官塘作业区一期顺利开港，落久水利枢纽、红花二线船闸加快建设，全国性综合交通枢纽建设扎实推进。胜利路高架桥、文昌路下穿通道、五岔路口改造基本完成；城市轨道交通前期工作进展顺利，白沙大桥、官塘大桥、莲花大道等项目加快推进，智慧城市、海绵城市、“城市双修”%和地下综合管廊试点有效开展。

　　——供给侧结构性改革成效明显。认真落实广西降成本“新28条”措施和柳州供给侧改革“1+5”政策&，扎实推进“三去一降一补”工作'，全年降低企业用电成本1.53亿元，为企业节约融资成本2.5亿元。打好“营改增”、降低涉企收费等“组合拳”，全年减轻企业、个人税费负担73.2亿元。

　　——人民生活水平不断提升。城镇居民和农村居民人均可支配收入分别实现32840元和12210元，增长8.5%和10%。财政投入民生保障的支出进一步提高，占财政总支出的80%。全面完成自治区、柳州市为民办实事任务。初步核验，年内69个贫困村脱贫出列、实现脱贫54766人，圆满完成自治区下达的年度任务。全市就业创业、社会保障、教育文化、医疗卫生等保障水平进一步提高，市民获得感不断提升。

　　一年来，我们做了以下工作：

　　持续调整优化产业结构。工业加快转型升级。汽车全产业链布局进一步完善，汽车产量超过250万辆，增长1.2%，上汽通用五菱自动挡、东风柳汽“双T”动力等新车型推向市场，宝骏E100成功上市，柳东商用车基地生产线竣工投产，加快汽车机械零部件再升级改造，汽车工业产值2517.8亿元，增长4.7%。钢铁工业加快产品结构调整，完成产值867.3亿元，增长14.3%，品种钢产量比重达63.5%，柳钢实现利润43亿元，创历史最好水平。机械工业加快技术创新和智能化生产，完成产值406.7亿元，增长19%，柳工加快智能化工厂改造升级，欧维姆智能制造能力持续提升。全市传统产业振兴步伐加快，鱼峰水泥生产规模实现翻番，熟料产能增量位居全国首位。战略性新兴产业加快培育，新能源汽车形成政企联动“柳州模式”，智能交通产业园一期工程、广西首家装配式建筑产业园开工建设。国家小微企业创业创新城市示范工作成效明显，柳州科技型中小企业孵化园被评为国家级小型微型企业创业创新示范基地。柳州被确定为国家知识产权试点城市，高新区通过国家知识产权试点园区验收。粤桂黔高铁经济带合作试验区广西园加快建设。北部生态新区规划和基础设施建设、产业招商等工作稳步推进。现代服务业优质高效发展。宁铁柳州汽车工业物流园等5家集聚区被认定为自治区级现代服务业集聚区，工业设计、检验检测等生产性服务业快速发展。全市新增182家限额（规模）以上服务业企业，达1288家。“互联网+”行动计划深入实施，袋装螺蛳粉销量位居全国米粉类电商产品第一，广西糖网获批国家电子商务示范企业，全市电商交易额798.7亿元，增长32%。开展遏制房价过快上涨等调控工作，房地产市场发展态势良好。旅游产业快速发展，三江成为广西特色旅游名县，融水争创广西特色旅游名县已获公示，百里柳江景区荣获全国体育旅游示范基地，国家4A级旅游景区增至24家，全市旅游总收入442亿元，增长26%。农业现代化水平稳步提高。新增6个自治区级、6个市级现代特色农业示范区。粮食产量保持稳定，水果、桑蚕、茶叶、水产畜牧养殖等产业规模化生产，11万亩“双高”基地建设扎实推进。农民专业合作社增至2500家。柳城华侨农场被认定为第七批全国“一村一品”示范村镇。全市农林牧渔业总产值320.9亿元，增长3.5%；一产增加值190.1亿元，增长3.5%。主要农作物耕种收机械化作业水平达59.5%。

　　全面打好精准脱贫攻坚战。全年投入财政专项扶贫资金15.99亿元，增长56.2%。出台精准脱贫12个实施方案，建成20户以上自然村（屯）道路1071.6公里，解决1.6万贫困人口饮水问题，完成5573户贫困户危房改造，基本解决贫困村宽带信息网络全覆盖和贫困户生产生活用电困难。易地扶贫搬迁扎实推进，共搬迁安置3.73万建档立卡贫困人口。湛江—柳州“携手奔小康”扶贫协作行动有效开展。为每个贫困村注入50万元发展村集体经济，209个贫困村实现产业项目全覆盖。

　　美丽柳州建设取得新成果。在全区率先开展全域空间规划试点工作，全面启动全国城市总体规划修编试点，城市发展顶层规划不断完善。国家新型城镇化综合试点、国家建制镇示范、广西百镇建设示范扎实开展。实施“城市管理提升年大行动”，城市管理信息中心被评为第一批国家绿色数据中心。扎实开展城市噪声污染专项整治和地质灾害治理。全面完成年度征地拆迁任务，实施清理整治违法建设“飓风行动”，拆除违法建筑面积458.3万平方米，圆满完成清除违法建设现有存量50%的目标。生态文明建设深入推进，国家第三批低碳城市试点深入实施，市区空气质量优良率为84.4%，柳江河饮用水保护河段保持国家地表水Ⅲ类以上水质标准。“花园城市”2.0版加快建设，全市森林覆盖率65%，城市建成区绿化覆盖率43.9%。开展“美丽柳州·宜居乡村”建设，建成30个生态乡镇、13个生态村，产业富民、基础便民等工作成效明显。

　　推进改革攻坚和扩大开放合作。深化“八个一”综合改革(，市级44个部门单位全部公布权责清单。“放管服”工作)扎实开展，“照证一窗通”“政银合作”服务效果良好，全面落实不动产统一登记制度。深化国企国资改革，建立外派监事会制度，国有资产总量达5500亿元，增长13.7%。非公有制经济健康发展，非公企业达23.2万家。深化投融资体制改革，引入社会资本61.7亿元参与城市建设，新增金融融资总量516.1亿元，增长7.2%；银行各项存贷款余额分别增长12.9%和9%，全市金融机构增至91家，上市及新三板挂牌企业达22家。商事制度改革扎实推进，年内新登记注册市场主体4.41万户，增长33.5%。基本完成农村土地承包经营权确权登记颁证工作，农村土地流转率24.2%。积极融入国家“一带一路”、珠江—西江经济带建设，成功举办第七届中国—东盟汽车工业博览会。企业“走出去”步伐加快，柳工海外客户数超过1.8万家，上汽通用五菱印尼工厂建成投产。粤桂黔高铁经济带合作项目扎实推进。柳州白莲机场临时航空口岸获批。全年完成外贸进出口总额163亿元，增长20%；引进市外境内到位资金849.2亿元，实际利用外资6.25亿美元。

　　持续改善社会民生。“大众创业、万众创新”深入开展，城镇新增就业6.93万人，农村劳动力转移就业7.66万人，城镇登记失业率3.52%。民生保障不断加强，全市基本养老保险、基本医疗保险参保率持续提升，城乡低保标准再次提高，实现农村与城镇居民医保同城同等待遇。投入3.3亿元建设102个社区服务场所。为北部三县25.6万户农村住房统一购买保险。保障性住房建设任务超额完成。提前三年实现县域义务教育基本均衡发展目标，率先成为广西所有县区全部通过国检的地级市。市区新建、改建学校31所，三年累计新增学位7.4万个。新增自治区级多元普惠幼儿园119所、新建儿童家园172个。普通高中高考一本上线率达20%，位居全区第一。现代职教全国学徒制试点稳步推进。文体事业繁荣发展，民族歌舞剧《白莲》、舞蹈诗《侗》、民族音画《八桂大歌》国内外巡演60多场，工业博物馆被评为国家工业遗产旅游基地，“三馆一站”*免费开放，卡乐星球欢乐世界建成开放。柳州籍体育健儿在全运会再创佳绩，成功举办2017年柳州世界铁人三项赛暨铁人世锦赛资格赛、2017国际水上狂欢节和环广西公路自行车世界巡回赛柳州段等赛事。医疗卫生事业加快发展，顺利通过国家卫生城市复审，全国中医药传承创新工程落户柳州，中医院东院投入使用，公立医院综合改革成效明显并获国务院表彰。依法实施全面两孩政策，妇幼健康服务质量持续提升，河东老人公寓等养老项目建成使用。“平安柳州”深入推进，公安“四项建设”+效果明显，矛盾纠纷化解有效开展，食品安全、安全生产责任体系进一步完善。积极创建全国文明城市，开展民族团结进步创建活动。国防动员准备和后备力量建设扎实推进，军民双拥共建深度融合，军政军民团结更加巩固。

　　主动接受人大法律监督、工作监督和政协民主监督。坚决执行市人大及其常委会决议决定，提请市人大常委会审议地方性法规2项，制定政府规章2项，认真贯彻实施首部实体性法规《柳州市莲花山保护条例》。办理人大代表议案3件、建议95件和政协提案199件，市人大代表议案建议、市政协委员提案办结率和满意率均达100%。

　　过去一年，市政府以高度政治自觉学习宣传贯彻党的十九大精神，深入开展“两学一做”学习教育，牢固树立“四个意识”，切实把党的十九大精神落实到经济社会发展各方面。市政府及各工作部门认真开展落实中央八项规定精神的自查自纠和“回头看”工作，抓好自治区党委第一巡视组巡视反馈意见整改，根据市委“作风整治年”“纪律教育年”“能力提升年”的工作要求，部署开展政府系统作风建设年活动，进一步规范了各项工作制度，加强行政监察和审计监督，狠抓督查落实，政府执行力和公信力不断提升。一年来，全市人事、编制、外事、侨务、税务、物价、统计、审计、国家安全、决策咨询、档案、地方志、气象、水库移民、供销、民族宗教、老龄、妇女儿童、残疾人事业、防汛抗旱、防震减灾、社会科学等各项工作也都取得新的成绩，作出了新的贡献。

　　在总结成绩的同时，我们也清醒地看到面临的问题和困难，主要是：传统产业对经济增长的拉动作用有所减弱，新兴产业集聚度不高，对经济增长贡献率较低，特别是中小企业自主创新能力不强、融资难度较大，产业转型升级任重道远；区域发展不平衡不充分仍很明显，民生保障工作有待进一步加强，脱贫攻坚、城市管理、社会治理等任务还十分艰巨。对于这些问题，我们将高度重视，采取有力措施，努力在实践中把各项工作做得更好。

　　各位代表！过去一年成绩来之不易，这是党中央、国务院，自治区党委、政府和市委坚强领导的结果，是市人大、市政协大力支持的结果，是全市广大干部群众攻坚克难、团结奋斗的结果。在此，我代表市人民政府，向全市各族人民，向驻柳人民解放军、武警驻柳部队官兵，向各民主党派、工商联、人民团体和海内外所有关心、支持柳州发展的各界人士，致以崇高的敬意和衷心的感谢！

　　2018年主要工作思路与预期目标

　　今年，是贯彻党的十九大精神的开局之年，是改革开放40周年，是决胜全面建成小康社会、实施“十三五”规划承上启下的关键一年，也是自治区成立60周年。刚刚胜利闭幕的中共柳州市第十二届代表大会第三次会议，市委郑俊康书记代表市委所作的工作报告紧紧围绕贯彻落实党的十九大精神，以习近平新时代中国特色社会主义思想为指引，明确了今年的发展目标、战略任务和重大举措，我们要齐心协力，认真抓好贯彻落实。

　　新时代、新思想、新征程，要有新的作为。新的一年，我们必须深入学习贯彻党的十九大精神，高举习近平新时代中国特色社会主义思想伟大旗帜，坚决维护习近平总书记的核心地位，统一思想、凝聚力量，全面推动柳州发展；我们必须紧扣广西“三大定位”新使命和“五个扎实”新要求，全力推进“三大建设”,、营造“三大生态”，积极谋划柳州发展新格局；我们必须以推进供给侧结构性改革为主线，把产业转型升级、战略性新兴产业培育作为主攻方向，把发展经济的着力点放在实体经济上，加快构建现代产业体系；我们必须高度重视投资和项目建设，全力以赴抓投资推项目，为经济社会发展不断注入新动力；我们必须用心做好城市工作，补好基础短板、做足绿色文章、打好生态品牌，提升城市品质；我们必须以实现精准脱贫为核心任务，切实保障和改善民生，让柳州人民的获得感、幸福感、安全感更加充实、更有保障、更可持续。

　　2018年工作的总体要求是：深入学习宣传贯彻党的十九大精神，以习近平新时代中国特色社会主义思想为指导，全面贯彻党中央、国务院、自治区和市委的决策部署，坚持稳中求进工作总基调，按照高质量发展的要求，以推进供给侧结构性改革为主线，深入实施“实业兴市，开放强柳”战略，抓重点、补短板、强弱项，践行“五个扎实”要求，着力解决发展不平衡不充分问题，坚决打好产业转型升级、基础设施建设和精准脱贫三大攻坚战，全力建设国际通道的核心枢纽、战略支点的实业引擎、重要门户的开放高地，持续营造风清气正的政治生态、团结和谐的社会生态、山清水秀的自然生态，为实现“率先建成”和“打造龙头”双目标持续夯实基础。

　　2018年柳州经济社会发展的主要预期目标是：地区生产总值增长7.5%左右，规模以上工业增加值增长6.5%，固定资产投资增长15%，财政收入增长8%，社会消费品零售总额增长10%，城镇居民人均可支配收入增长7.5%，农村居民人均可支配收入增长9.5%。城镇登记失业率和单位生产总值能耗、主要污染物排放总量控制在自治区下达指标以内。

　　以上预期目标，基于以下几方面考虑：一是充分体现高质量发展要求。增长7.5%左右的目标，是在广泛征求意见、科学测算、全面权衡的基础上确定的，既遵循经济增速高于国家而且不低于自治区增速的原则，又把聚焦点转到提质增效上来，符合柳州发展的阶段性特征。同时，自治区制定今年经济增长7%—7.5%的目标，并且要求“条件好的市在确保质量效益的前提下争取高于全区水平”。柳州作为广西最大的工业城市和经济总量第二大城市，按照自治区要求应当主动作为、多作贡献。二是充分体现稳中求进的总基调。增长7.5%左右是一个区间预期目标，符合稳中求进的要求。从当前形势看，实现7.5%左右的增速确实有难度，但并非没有希望，综合各方面考虑，柳州有基础、有条件实现企稳向好发展，这需要付出艰苦努力，更要有“跳起来摘桃子”的勇气。三是充分考虑与“十三五”发展目标的衔接。“十三五”规划提出全市地区生产总值年均增长8%的目标，并明确柳州要在全区率先全面建成小康社会，这就需要在“十三五”期后三年经济保持一定的中高速增长。我们坚信，只要全市上下坚定信心，团结一致，克难攻坚，就一定能够走出一条高质量的发展道路。

　　2018年主要工作任务

　　围绕今年的目标任务，我们将重点抓好以下几个方面工作：

　　一、提质增效振兴实体经济，加快建设战略支点的实业引擎

　　全力打好产业转型升级攻坚战。没有产业的转型升级，就没有经济的高质量发展。继续深入贯彻实施《中国制造2025》，努力打造国家重要的先进制造业基地。扎实推进工业提质增效转型升级工程，建设100个重点工业项目。汽车工业向中高端车型升级，建设上汽通用五菱技术中心试验室、耐世特二期等项目，竣工投产联合电子二期等一批项目。机械工业深入实施“二次创业”，大力发展工业机器人产业，继续推进柳工挖掘机装配智能工厂、欧维姆智能制造等项目建设。深入实施汽车机械零部件再升级改造，构建零部件基础配套体系、新能源汽车零部件本地配套体系，促进零部件产业与整车协同发展。钢铁工业持续优化产业结构，加快推进柳钢工业绿色项目建设，形成钢铁主业与多元产业协同发展新格局。积极运用信息技术和先进适用技术改造提升建材、化工、食品、纺织等传统产业，促进传统产业焕发新活力、形成新优势。

　　大力发展战略性新兴产业。优先培育城市轨道交通、智慧电网、装配式建筑、工业设计、工业机器人、工业大数据产业，提升壮大新能源汽车、高端装备制造、电子信息、新材料、节能环保、生物与制药产业，构建战略性新兴产业发展新体系。推动新能源汽车产业化发展，打造电机、电池、电控三大核心零部件产业链集群，年内新能源汽车产量达5万台以上。大力发展城市轨道交通产业，建设智能交通产业园，实现轨道交通PC-轨道梁预制场一期项目竣工投产。加快发展装配式建筑产业，推进广西建工柳州装配式建筑产业园、柳钢钢结构装配式建筑产业现代化基地建设，实现装配式建筑产业园PC预制基地竣工投产，年内装配式建筑占新建建筑比例达8%以上。继续扶持培育30家战略性新兴产业骨干企业，力争战略性新兴产业产值比重达13%以上。

　　加快建设创新型城市。实施城市转型升级质量发展导则，开展全领域质量提升行动，大力推进全国质量强市示范城市建设。进一步完善知识产权管理体系，加快推进柳州国家知识产权试点城市建设，协同争创国家自主创新示范区，努力创建国家创新型城市。建设国家信息中心大数据工业应用（柳州）基地，确保完成国家汽车质量监督检验中心（柳州）一期验收申报。实施柳东国际合作技术中心、新能源汽车检测平台等项目，培育300家以上高新技术企业。深入开展“两创”示范工作，促进“两化”深度融合，发展智能工厂、数字化车间、工业互联网，推广工业云服务创新应用，加快新旧动能接续转换。

　　深入实施人才强市战略。深化人才优先发展体制改革，大力开展“优秀人才接触柳州、优秀人才创业柳州、优秀人才服务柳州”三大活动，制定引进“高精尖缺”人才的政策措施，建设人才公寓，打造具有区域竞争优势的人才发展环境，吸引高层次创新创业人才和急需紧缺人才向柳州聚集。

　　不断提高园区产业集聚与承载能力。柳东新区重点推进东风柳汽商用车基地二期、玲珑轮胎二期、智能交通产业园等项目竣工投产，开发江滨片区、中欧片区、花岭片西部路网，完善新区供水供电供气、通讯管网等配套设施。北部生态新区加快推进广西（柳州）智能制造城建设，发展智慧电网、工业机器人、工业设计和工业大数据等产业，推动智能制造产业要素集聚发展。做好阳和工业新区申报国家级经济技术开发区、鹿寨经济开发区申报自治区级高新技术产业开发区工作，启动柳江高新技术开发区规划建设，提高河西高新技术产业开发区、柳北工业区、洛维工业集中区、柳江新兴工业区、柳城工业区等园区承载能力，年内园区产业集中度达58%以上。

　　二、实施乡村振兴战略，全力构建特色现代农业产业体系

　　加快城乡一体化发展。深入实施基础设施建设三年行动计划，制定乡村振兴战略规划和实施方案。加快融水至从江高速公路一期工程（融水至安太段）前期工作，争取年内开工。实施“四好农村路”建设，支持融水县争创国家级“四好农村路”示范县，支持柳城县、鹿寨县争创自治区级“四好农村路”示范县。建设柳州国家新型城镇化综合试点信息应用平台，统筹城镇规划、建设与管理，打造产业特色鲜明、生态环境优美、功能配套齐全的特色小镇。大力推进柳城—融安—融水—三江北部绿色生态工业带开发，完善“一圈一带四轴”.新型产城融合体系。

　　发展特色优质安全高效现代农业。实施现代特色农业示范区增点扩面提质升级三年行动计划，推进“三区三园一体”/建设，创建国家级现代农业产业园。实施“产业富民”行动，巩固粮食生产，大力发展优质水果、高效桑茧、名优茶叶等特色产业，建设畜禽标准化养殖场、水产健康养殖场，完成16.7万亩“双高”基地建设任务。推进农产品精深加工园建设，培育壮大农业产业龙头企业，新增100家新型农业经营主体。开展“互联网+现代农业”行动，实现电子商务进农村综合示范县项目全覆盖。加强农产品质量安全监督，促进农业生产绿色化、标准化、品牌化。

　　持续改善农村人居环境。深入推进“宜居乡村”建设，完善城镇供水、污水和垃圾处理设施，保护和发展民族特色村寨。实施“服务惠民”“基础便民”行动，推进乡乡通二级（三级）公路建设，实现农村公路“村村通”。深入开展村容村貌整治，推行农村生活垃圾分类收集处理，提升改造融水、三江连片木结构少数民族村寨防火设施。分类指导特色产业、传统文化、自然生态村落建设，打造12个重点示范村，实现“一村一景、一村一品、一村一韵”。

　　大力发展县域经济。实施县域产业发展、基础设施和公共服务设施能力提升3个专项行动计划，重点支持县域和乡村建设。柳江区打造“中国·百朋莲花特色小镇”，建设新城区城市综合体等项目，形成特色鲜明、产业互动、功能完善的要素集聚承载地。柳城县在产业发展上与市区紧密对接、联动发展，开发凤山特色小镇，建设现代生态农业种养示范园。鹿寨县加快与市区同城化发展，建设石墨烯新材料产业基地，大力发展热能热电新材料、新能源产业。融安县推进金桔等特色农业产业升级，打造广西香杉产业中心，加快生态工业集群化发展。融水县全面启动全域旅游重点基础设施大会战，加快贝江特色小镇、元宝山生态旅游等项目建设，发展培育竹木精深加工、健康食品等产业。三江县启动大洲岛文化旅游综合体、仙源健康产业园等项目建设，加快打造成为桂湘黔三省区交界处旅游集散中心、旅游目的地。

　　三、实施服务业振兴行动，全面提升现代服务业发展水平

　　推动生产性服务业向专业化和价值链高端延伸。全力推进现代物流、汽车服务、专业市场、电子商务四大产业集群，加快打造广西生产性服务业发展高地。大力发展枢纽经济，筹划高铁航空物流基地，推进上汽通用五菱汽车工业物流园、官塘多式联运物流园、传化公路港、毅德商贸物流城等集聚区建设，加快食品冷链物流产业园、海吉星农产品批发市场二期、三江茶叶交易中心等项目的建设，努力打造成为中国与东盟双向往来的物流中转基地和加工贸易基地。

　　推动生活性服务业向精细化高品质转变。发展创意设计、节庆会展、文体休闲等高端服务业，提高生活性服务业发展品质。全力打造“电商柳州”，深化与电商企业合作，支持传统商贸企业转型升级，融合线上线下推动零售业发展。建设螺蛳粉特色小镇，申报柳州螺蛳粉地理商标，加快培育螺蛳粉全产业链。实施农贸市场升级改造，制定集贸市场建设管理办法。加快华润商业中心、三胞广场、园博园友邻汇等城市商业综合体建设，完善城市商圈功能。坚持房子是用来住的、不是用来炒的定位，抓好房地产市场分类调控，形成促进房地产市场健康发展的长效机制。

　　增强金融服务实体经济功能。积极规范发展多层次资本市场，着力打造区域性产业金融服务中心。推广政府和社会资本合作模式，利用城镇化基金、企业债券、农业产业基金等方式拓宽融资渠道。做好中小微企业金融服务，进一步降低企业融资成本。加快企业上市步伐，支持花红药业、双英股份、欧维姆、方盛集团等企业申报IPO0。坚决打击和处置非法集资，守住不发生区域性金融风险的底线，营造诚信安全金融生态。

　　加快全域旅游产业规划与建设。全力支持三江、融水创建“国家全域旅游示范区”，继续推进融水、鹿寨创建广西特色旅游名县，谋划打造柳城凤山旅游区和优质养生区。集中市级财力和资源支持承办旅游产业发展大会的县区建设优质旅游项目。完善旅游基础设施和公共服务功能，推动百里柳江、程阳八寨争创国家5A级旅游景区，加快实现旅游景区提档升级。

　　四、坚决打赢精准脱贫攻坚战，做到脱真贫、真脱贫

　　扎实推进精准扶贫、精准脱贫。全力开展“七个一批”“十大行动”1，年内确保实现减少5万以上农村贫困人口、50个以上贫困村脱贫，力争融安县脱贫摘帽。重点抓好深度贫困地区脱贫攻坚，落实重大扶贫工程、帮扶到村到户等措施，大力推进特色产业、基础设施建设大会战、易地扶贫搬迁等工作。继续开展湛江—柳州“携手奔小康”扶贫协作行动，推动社会扶贫资源与扶贫需求精准对接，不断增强扶贫工作合力。

　　推进重点领域集中攻坚。抓好产业扶贫，确保扶贫开发任务县分别发展2—5个特色优势产业、172个深度贫困村每村发展1个以上主导产业，构建“县有扶贫支柱产业、村有扶贫主导产业、户有增收致富项目”的产业扶贫新格局。实施发展壮大村级集体经济三年行动计划，安排财政资金支持155个贫困村、190个非贫困村发展集体经济。开展贫困地区基础设施建设大会战，实现贫困村20户以上自然村（屯）硬化路全覆盖，完成5700户贫困户危房改造，对114个贫困村（屯）老旧供电网络进行更新改造。扎实推进易地扶贫搬迁，确保1.56万人实现易地搬迁入住。

　　完善精准脱贫工作机制。强化党政一把手负总责，健全结对帮扶到村到户“三包”机制。坚持督查、通报、协调制度，严格抓好绩效考核、实绩评估工作。调动基层积极性，发挥群众主观能动性，克服“干部干、群众看”现象。整合各类扶贫资金，提高扶贫资金使用的精准性、有效性，加大扶贫资金使用的审计、监察力度，确保扶贫资金到村、到户、到人。强化脱贫攻坚信息动态精准管理，落实脱贫摘帽退出机制，严防“数字脱贫”“虚假脱贫”。

　　五、持续打好基础设施建设攻坚战，进一步扩大有效投资

　　狠抓重大项目建设。今天的投资就是明天的动能，今天的项目就是明天的结构。从今年开始，实施重大项目建设三年行动计划，以项目建设促进稳增长、调结构、惠民生、补短板。继续抓好自治区层面、市级层面、西江经济带基础设施大会战、自治区成立60周年等项目建设，今年实施541个重大项目，计划投资823.4亿元。其中，新开工柳州动车组存车场等212个项目，年度计划投资264.1亿元；续建西外环工程等207个项目，年度计划投资401.4亿元；竣工东风柳汽迁建10万辆商用车等122个项目，年度计划投资157.9亿元。实施基础设施、产业发展、生态环保、民生保障、社会管理等5个领域城市基本建设项目1028项，年度计划投资568.9亿元，以项目建设实实在在的成效夯实稳增长基础。

　　扎实做好项目建设要素保障。积极搭建“政府、金融机构、企业”多方合作平台，加强政银企沟通协调，促进金融资源与项目有效对接，确保项目建设筹融资有效落实。强化用地保障，加大征地拆迁力度，坚持节约集约利用土地，盘活存量、用好增量。着力改善投资环境，继续实行领导干部联系重点项目制度，精简行政审批事项，提高项目审批效率，积极为投资和项目建设提供优质高效服务。

　　六、增强城市综合服务功能，加快建设国际通道的核心枢纽

　　建设内联外通交通新体系。继续推进全国性综合交通枢纽建设，加快打造面向东盟、服务“一带一路”、服务西南中南、融入粤港澳大湾区的重要枢纽。继续推进柳州至肇庆、涪陵至柳州铁路以及柳州至三江、柳州至河池、柳州至贺州等城际铁路的前期工作。抓紧柳州至南宁改扩建、桂林至柳城、融水至河池、柳州经合山至南宁、阳朔至鹿寨等高速公路项目建设。推进柳州港鹧鸪江、江口等作业区建设，加快红花二线船闸改造，全力推进落久水利枢纽建设，力争开工建设洋溪、梅林枢纽工程。推进机场远距离二跑道前期工作。争取城市轨道交通规划通过审批，年内实现示范线车辆上线。加快火车站城市综合交通配套工程建设，确保火车站站房及站前广场顺利完工。规划建设官塘码头铁路专线，形成多式联运。

　　持续完善城市基础设施。继续推进全域空间规划试点工作，深入编制新一版城市总体规划，扎实开展新一轮土地利用总体规划修编。加快“一主三新”2城市建设，柳东新区完善城市基础设施配套，加大国家产城融合示范区建设力度；优化柳江区发展规划，大力推进撤县设区城市基础设施项目建设，提升新城区承载能力；加快北部生态新区发展，全力推进湿地公园、“三纵三横”城市主干道和骨干光纤网络建设，加快打造成为新型城镇化建设示范新区和智慧城市新区。完善城市基础设施，开工建设凤凰岭大桥，建成白沙大桥、官塘大桥，实现官塘核心区水系补水综合整治等一批项目竣工。加快推进地下综合管廊、海绵城市、智慧城市建设和“城市双修”工作。实施旧城改造、城中村改造和历史文化街区保护整治，开展老旧小区、“三无”小区综合改造。进一步完善市政道路、慢行交通系统、智能停车场、汽车充电桩等公共设施。每个城区建设一个体育公园，进一步完善城市服务功能配套，逐步实现“15分钟步行生活圈”3。

　　提升城市治理现代化水平。强化城市精细化管理，推行“721工作法”4，全面推进政府热线、社会治安、城管综合执法等信息网络融合，深入开展清理整治违法建设“飓风行动”、城市道路扬尘专项治理、地质灾害防治，加大铁路及公路沿线、柳江沿岸、城郊结合部、城市进出口道路整治力度，让城市更有序、更安全、更干净。

　　七、着力提升绿色发展水平，构建山清水秀的自然生态

　　加快发展生态经济。紧扣北部生态新区“生态”特色，构建完整规划体系，加快产业腾笼换鸟，大力发展智能制造、生态环保等产业，打造生态产业链。推进国家循环经济示范城市和低碳城市建设，培育壮大节能环保、新型建材、新一代信息技术、新材料等新兴产业，大力发展生物医药、健康养生等生态服务业。加快实施鹿寨县经济开发区国家级循环化改造、广西柳州汽车城电镀工业园等项目建设。

　　持续改善生态环境。坚决打好污染防治攻坚战，落实大气、水、土壤污染防治行动计划。针对市区空气质量优良率下降问题，制定治理方案，采取有力措施，切实提升市区空气质量。完成竹鹅溪整治配套工程，开展黑臭水体整治效果评估，建立“长制久清”工作机制；严格落实“河长制”，健全河长信息管理体系。针对市民反映噪声扰民的突出问题，组织力量、下大力气、持之以恒抓好噪声污染专项治理，营造市区和谐安宁的生活环境，还市民一片宁静。开展城乡环境综合整治，加强莲花山生态保护。加快建设静脉产业园，推进生活垃圾焚烧处理工程，全面实施城市生活垃圾分类收集处理。继续推进“厕所革命”，新建（改建）一批旅游厕所。牢固树立“绿水青山就是金山银山”理念，深入推进“花园城市”2.0版建设，打造“紫荆花城”“春花秋水”品牌，确保顺利通过国家园林城市复核验收。深入开展生态文明建设示范工作，创建一批生态乡镇和生态县，不断提升生态发展水平。

　　八、以改革创新优化营商环境，加快建设重要门户的开放高地

　　深化重点领域改革。深入推进供给侧结构性改革，全面落实“1+5”政策文件。抓好国企国资改革，进一步理顺市属国有企业治理结构、资产结构，优化经营方式和管理手段，提高经营性资产比例，增强九大集团公司“造血”功能和运营能力。深化“一枚印章管审批”改革，开展县区相对集中行政许可权改革试点。深入实施商事制度改革，进一步降低市场准入门槛。推进国家监察体制改革试点工作，做好过渡期各项政策和工作的有效衔接。完善财政资金统筹管理机制，加强政府债务限额管理。积极发展混合所有制经济，抓好促进非公有制经济发展政策措施落实。推进集体林权制度改革，探索建设农村产权流转交易市场，进一步健全农业社会化服务体系。统筹实施文化、教育、医疗卫生、社会保障、食品药品等领域改革。

　　大力实施开放带动战略。积极融入国家“一带一路”、珠江—西江经济带、粤桂黔高铁经济带合作与建设。主动对接粤港澳大湾区，强化互联互通。促进柳来河规划、交通、产业一体化发展。加快粤桂黔高铁经济带合作试验区广西园建设。实施新一轮加工贸易倍增计划，争取柳州保税物流中心（B型）启动建设。支持上汽通用五菱、柳工、欧维姆等企业海外布局发展。抓住中新互联互通南向通道建设的机遇，加快形成陆海内外联动、东西双向互济的开放新格局。

　　开展优化营商环境集中行动。针对国家统计局柳州调查队反映柳州投资环境和投资满意度下降问题，制定优化营商环境专项整改措施和评价机制，切实提升投资环境满意度，提振投资者和企业家投资信心，让更多的先进制造业、生产要素、人才集聚柳州。深入实施招商引资三年行动计划，加大对世界500强、中国500强及重点民企的招商力度，加强与珠三角、长三角等发达地区合作，年内实现市外境内到位资金增长7%以上、实际利用外资增长10%。

　　九、着力改善民生福祉，不断满足人民日益增长的美好生活需要

　　推进社会保障稳步发展。坚持以人民为中心的发展思想，着力补齐民生短板，扎实做好为民办实事工作，积极营造团结和谐的社会生态。深入实施城乡居民收入倍增计划，持续开展“大众创业、万众创新”，不断拓宽高校毕业生、农民工等群体就业创业渠道。实施全民参保计划，深化医疗保险支付方式改革，完善基本医疗保险制度。进一步提高城乡最低生活保障标准，实现农村低保制度与扶贫开发政策有效衔接。做好农村“三留守”人员5关心关爱工作，提高孤儿基本生活最低养育标准。支持社会福利和慈善事业发展，大力发展老龄事业。推进医养结合，建设养老服务业综合改革试验区。全力做好保障性安居工程建设，实施新一轮棚户区改造，年内完成1.2万套保障性住房改造及新建任务。

　　推进教育事业优先发展。实施教育3个“三年行动计划”6，新建柳江新高中等一批中小学、幼儿园项目，确保铁一中柳东校区高中部、市一中迁建等学校2018年秋季招生办学。提高普惠性幼儿园覆盖率，力争学前三年毛入园率达85%。推动城乡义务教育一体化发展，完善集团化办学学区制管理、县属轮教等工作机制，办好特殊教育、网络教育，稳定偏远贫困乡村教师队伍。推进普及高中阶段教育，力争高中阶段毛入学率达85%。推动广西科技大学鹿山学院向应用型本科院校升级，积极筹建柳州理工学院。建设广西现代职业教育改革发展示范区、广西职业技能公共实训基地，深入开展全国学徒制试点，加快职业教育国际化发展。

　　推进医疗卫生事业持续发展。实施全民健康素养促进行动，加快健康柳州建设。认真落实国家卫生城市复审意见的整改，提高卫生城市建设水平。深化城市公立医院综合改革，完善医院现代化管理制度，加快工人医院搬迁、国家中医药传承创新工程等建设。大力实施市区医疗卫生基础设施提升三年行动计划，整体提升市区公立医院设施和服务水平。做好紧密型医联体建设，扩大县域医共体建设范围，提升基层医疗卫生机构服务能力。完善分级诊疗制度，健全药品供应保障机制。支持妇女儿童事业发展，加强疾病预防、残疾人康复等工作。继续为700名生活困难白内障患者提供免费复明手术。实施全面两孩政策，推动人口长期均衡发展。

　　推进文体事业繁荣发展。加快文化强市建设，精心筹备纪念改革开放40周年、庆祝自治区成立60周年重大文化活动，继续开展《侗》《白莲》《八桂大歌》等精品剧目国内外巡演，用艺术讲好柳州故事。加快柳东新区文化广场、图书馆、城市档案中心等建设。完成第三批国家公共文化服务体系示范项目创建。做好历史文化遗产传承利用，推进黔湘桂三地侗族村寨联合申遗，建成并开放白莲洞博物馆。加快文化产业项目建设，促进文化产业与旅游、科技、体育等融合发展。实施全民健身计划，认真组织筹办国际水上狂欢节、柳州奇石节和中国柳州世界铁人三项赛等重大活动赛事。继续争创全国文明城市，广泛开展精神文明创建活动，弘扬社会主义核心价值，倡导柳州精神。

　　推进社会治理稳定发展。强化“平安柳州”建设，深入实施公共安全视频监控联网工程，依法打击涉黑恶势力。加强社区建设与网格化管理，进一步畅通信访渠道，做好“三大纠纷”调处。严格落实安全生产责任制，推进“互联网+安全生产”信息化建设，坚决遏制重特大事故发生。积极创建广西食品安全城市（县），严格执行食品安全党政同责规定。深入开展民族团结进步创建活动，促进民族团结、宗教和谐、社会稳定。

　　推进军民融合深度发展。深入开展国防教育，积极支持、服务国防和军队现代化建设。推动军民深度融合，依托长虹公司建设军民融合产业园。做好新一轮双拥模范城创建工作，全面落实优抚安置政策，巩固军政军民团结大好局面。

　　十、持之以恒加强政府自身建设，努力打造法治、高效、廉洁政府

　　扎实开展“不忘初心、牢记使命”主题教育。按照中央、自治区和市委的工作部署，深入学习贯彻党的十九大精神，切实用习近平新时代中国特色社会主义思想武装头脑，牢固树立“四个意识”特别是核心意识，扎实开展“不忘初心、牢记使命”主题教育，推动“两学一做”学习教育常态化制度化，不断提高执政能力和领导水平。

　　加强法治政府建设。坚持依法决策，推行重大行政决策预公开制度，完善权力清单和责任清单。推进行政复议相对集中制度改革，争取率先在广西成立行政复议局。提高政府立法质量，加强全民普法，强化执法监督，严格规范公正文明执法，做好政务信息公开、舆情处理回应工作，让权力在阳光下运行。

　　加强廉洁政府建设。认真落实党风廉政建设主体责任，履行 “一岗双责”工作职责，贯彻执行中央“八项规定”精神和“八项规定”实施细则，针对“表态多调门高、行动少落实差”问题开展专项整治，杜绝“议而不决、决而不施、施而无效”现象，驰而不息纠正“四风”，强化监督执纪问责，严肃查处违纪违法，努力营造风清气正的政治生态。继续开展“市长接待日”“市长企业接待日”和领导公开大接访等工作，密切与人民群众的血肉联系。进一步严格控制“三公”经费，加强公务用车、办公用房管理。开展对财政资金、政府投资项目等审计工作，健全重大项目推进落实督查机制。

　　各位代表，新的一年，政府将更加主动地接受市人大及其常委会的法律监督、工作监督，认真执行人大及常委会的决议，更加自觉地接受政协的民主监督，认真办理人大代表议案、建议和政协委员提案，广泛听取各民主党派、工商联、无党派人士和人民团体的意见建议，体现人民意愿、维护人民利益、回应人民期待。

　　各位代表、各位委员、同志们，新时代、新思想、新征程，催人奋进。让我们更加紧密地团结在以习近平同志为核心的党中央周围，在自治区党委、政府和市委的坚强领导下，不忘初心、牢记使命，全力推进“三大建设”、营造“三大生态”，为加快实现“率先建成”和“打造龙头”双目标，建设富强民主文明和谐美丽的柳州而努力奋斗！
